# Impulsiveness indirectly affects suicidal ideation through depression and simultaneously moderates the indirect effect: A moderated mediation path model

**DOI:** 10.3389/fpsyt.2022.913680

**Published:** 2022-07-27

**Authors:** Jingxuan Zhang, Xiaolin Zhang, Guoyu Yang, Zhengzhi Feng

**Affiliations:** ^1^Department of Medical Psychology, Army Medical University, Chongqing, China; ^2^Teaching Examination Centre, Army Medical University, Chongqing, China

**Keywords:** impulsiveness, depression, suicidal ideation, moderated mediation path model, indirect effect

## Abstract

**Objective:**

This study aims to investigate the indirect effect of impulsiveness on suicidal ideation through depression and the moderating effect of impulsiveness on the indirect effect in an integrated path model.

**Methods:**

Self-rating depression scale (SDS), Barratt impulsiveness scale-11th version (BIS-11), and self-rating idea of suicide scale (SIOSS) were applied. A moderated mediation path model was established including impulsiveness, depression, and suicidal ideation as observed variables.

**Results:**

The main results revealed that the moderated mediation path model fit well in describing the relationships among impulsiveness, depression, and suicidal ideation. The indirect effect of impulsiveness mediated by depression and the moderating effect of impulsiveness on suicidal ideation was significant. Multiple comparisons showed that the indirect effects under different conditions of impulsiveness had statistical differences. The higher the impulsiveness was, the stronger the predictive effect of depression on suicidal ideation was.

**Conclusions:**

The present study confirms that people who have impulsive traits are riskier to generate suicidal thoughts because they are more likely to suffer from depression and that people who are depressive have even higher risk to develop suicidal thoughts when they simultaneously have impulsive traits. In clinical and health care work, when considering depression to prevent suicidal ideation, impulsiveness needs to be monitored throughout the process of premorbid and onset stages of depression.

## Introduction

According to the newest worldwide statistics (1), about 9.2 persons per 100,000 died from suicide in 2019. Suicide is divided into three phases: suicidal ideation, suicide attempts and lethal suicide behavior (2). The prevalence rate of suicidal ideation, which ranges from 2.6 to 25.4% (3), is much higher than suicide attempts and lethal suicide behavior. It is thought to be an indispensable cognitive process before suicide actions take place (4, 5). Solano et al. found that the Internet searching behaviors of suicide-related information were positively correlated with the growth of suicide rates in the following three months (6). The Internet searching behaviors represent the existence of suicidal ideation (6). Therefore, Solano's study indicates that suicidal ideation is a preexisting factor of lethal suicide behavior. A large sample survey revealed that individuals with suicidal ideation were over four times more likely to commit suicide than whom without suicidal ideation (7). Accordingly, for suicide prevention, it is critical to detect, manage, and intervene in suicidal ideation.

As described in the diathesis-stress-model (8), vulnerable trait factors (diathesis) and unexpected life events [including subsequent negative/positive emotion and psychopaths, et al., namely, stress (9)] cause the onset of suicide behavior, specifically, suicidal ideation. Among the diathesis and stress, impulsiveness and depression have been widely studied, which are the two vital factors that confer high risk. However, it is inconsistent about how the two factors work together in suicidal cognitive process.

In some studies, impulsiveness and depression are two independent variables related to suicidal ideation (10–12). Another study constructed a linear regression model (13), finding that depression independently predicted suicidal ideation and that impulsiveness affected suicidal ideation through interacting with traumatic experience. It is inconsistent about whether impulsiveness can independently lead to suicidal ideation and not clear whether impulsiveness can interact with depression in predicting suicidal ideation.

The integrated motivational-volitional model holds that impulsiveness is an interacting variable with suicidal ideation in predicting suicide behavior, while does not mention the effect of impulsiveness on suicidal ideation nor its interaction with any psychopaths (5). One study showed that, when depression existed, impulsiveness could raise the risk of suicide behavior rather than suicidal ideation (14). It indicates that impulsiveness interacts with depression in the onset of suicide behavior but not suicidal ideation. However, according to Beck's cognitive model of suicide (4), vulnerability and psychiatric disturbance jointly contribute to suicide related thoughts at first, and then lead to suicide behavior. This is possibly because not every “impulsiveness” in previous studies is a kind of diathesis. Some of them may refer to a kind of state (15). It may confuse the medical or public health personnel in considering whether to deal with impulsiveness as a diathesis to monitor or as a state to intervene.

State impulsiveness is a process of fast decision or behavior without thinking (16). However, trait impulsiveness is a stable style of behavior as a personality (17). It is subjectively measured by questionnaires, usually based on Barratt's impulsiveness model (18) or UPPS (urgency-premeditation-perseverance-sensation-seeking) impulsiveness model (19). State impulsiveness is less likely to take effect together with depression among the cognitive process of suicide (5). Therefore, in the present study, we mainly paid attention to trait impulsiveness (called “impulsiveness” in the following text). According to the integrated motivational-volitional model, we hypothesize that (trait) impulsiveness interacts with depression in predicting suicidal ideation, which can be described by a simple moderation model with the moderating effect of impulsiveness on the path from depression to suicidal ideation. However, the simple moderation model does not consider the correlation between impulsiveness and depression which have been proved closely correlated in previous studies (20–25). Therefore, according to Beck's cognitive model (4), in which vulnerability influences suicidal cognitive process through psychiatric disturbance, we hypothesize that impulsiveness indirectly affects suicidal ideation through depression.

**Figure 1:**
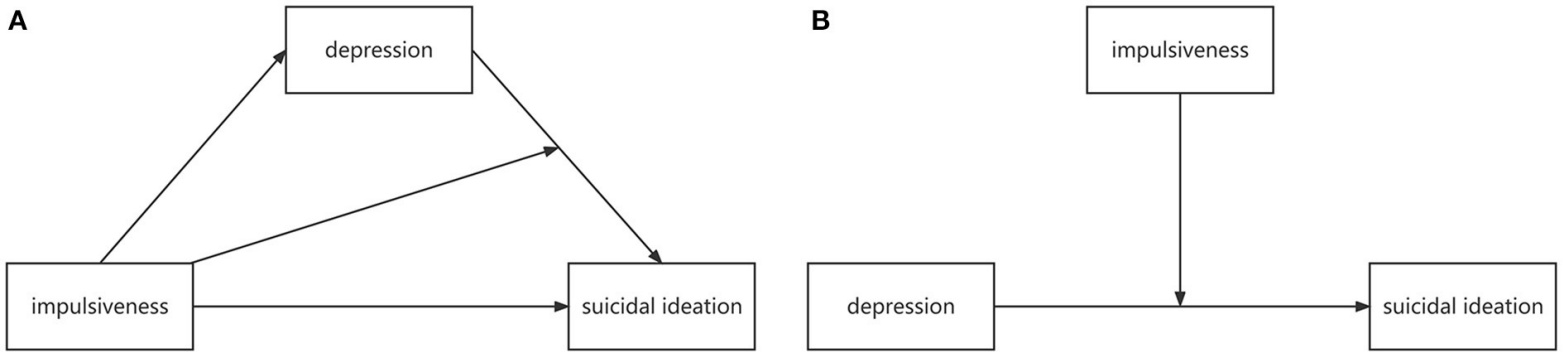
Conceptual diagrams of MODMEDPM **(A)** and SMODPM **(B)**. MODMEDPM—moderated mediation path model; SMODPM—simple moderation path model.

Combining these two hypotheses, we construct a moderated mediation path model (MODMEDPM). The whole hypothesis of this study is that impulsiveness indirectly affects suicidal ideation through depression and simultaneously moderates the indirect effect. To be more reliable, we take the simple moderation path model (SMODPM) as the alternative model for comparison. The conceptual diagrams of MODMEDPM and SMODPM are shown in Figure 1.

## Materials and methods

### Participants

We applied cluster sampling method to select a cohort who was healthy in mentality. To control the confounding effects, we only recruited two grades of students, including 196 freshman (40.8%) and 284 sophomore (59.2%). Because the junior, senior and interns studied in different hospitals, who were faced with mixed stressors which could be confounding variables. The psychological tests were conducted within one month. All the 480 participants were examined. The cohort included 245 females (51.0%) and 235 males (49.0%) aged 16–21 (18.66 ± 0.80). Other demographic characteristics, including grade, residence, only child status, and family structure, as well as descriptive statistics of variables were shown in Table 1. There were two participants who did not report family structure. They were rejected only when the family structure was included as a factor in the analysis.

**Table 1:** Demographic characteristics and descriptive statistics.

	**Total sample (*****n** =* **480)**	
**Variables (group, score)**	***n*** **(%)**	**Mean (SD)**
Gender (male, 1)	235 (49.0)	
Gender (female, 0)	245 (51.0)	
Grade (freshman, 1)	196 (40.8)	
Grade (sophomore, 0)	284 (59.2)	
Residence (urban, 1)	387 (80.6)	
Residence (rural, 0)	93 (19.4)	
Only child (1)	273 (56.9)	
Non-only child (0)	207 (43.1)	
Family structure (joint family, 1)	146 (30.4)	
Family structure (nuclear family, 2)	298 (62.1)	
Family structure (broken family, 3)	34 (7.1)	
Family structure (didn't report, null)	2 (0.4)	
Age	480 (100)	18.66 (0.80)
Impulsiveness-raw	480 (100)	72.50 (14.94)
Depression-raw	480 (100)	37.10 (9.06)
Suicidal ideation-raw	480 (100)	5.11 (4.00)
Impulsiveness-centered	480 (100)	0.00 (14.94)
Depression-centered	480 (100)	0.00 (9.06)
Suicidal ideation-centered	480 (100)	0.00 (4.00)

SD, standard deviation; “-raw”, the raw scores; “-centered”, the centered scores.

### Measures

#### Suicidal ideation

Suicidal ideation was assessed by the twenty-six-item Self-rating idea of suicide scale (SIOSS). This scale was developed in Chinese by Xia (26). The participants choose yes or no concerning whether they have symptoms or ideas described in the items. Higher sum scores imply stronger suicidal ideation. The SIOSS has good reliability and validity in the Chinese population (27–29). In the present study, this scale had acceptable reliability of internal consistency (Cronbach's alpha = 0.822).

#### Impulsiveness

The Barratt impulsiveness scale (SIOSS) consists of 30 items. It was originally developed by Ernest S. Barratt in 1959 (18). The 11th Chinese version of the BIS was revised under the context of Chinese culture and retained good reliability and validity (30). The BIS had fine internal consistency reliability (Cronbach's alpha = 0.909) in this study.

#### Depression

The Self-rating depression scale (SDS) was initially developed by William W. K. Zung in 1965 for evaluating depression in patients with depressive disorder (31). The scale consists of 20 items. It can also be used for preliminary screening in outpatient clinics (32), early detection of depression (33) or depressive state evaluation in the general population (34, 35). The Chinese version of the SDS has demonstrated good reliability and validity across various groups of people (36). Its internal consistency reliability was good (Cronbach's alpha = 0.879) in this study.

### Statistical analyses

The internal consistency reliability coefficients and the correlation coefficients of the variables were calculated in SPSS 20.0 (37). We also made the descriptive statistics for demographic variables in SPSS 20.0. Model fits and path analyses were conducted in Mplus 8.3 (38). The Mplus code for mediation and moderation models was developed by Stride et al. (39), according to Hayes' PROCESS macro program documentation (40). We modified the Mplus code for adjusting the purpose of the present study.

**Figure 2:**
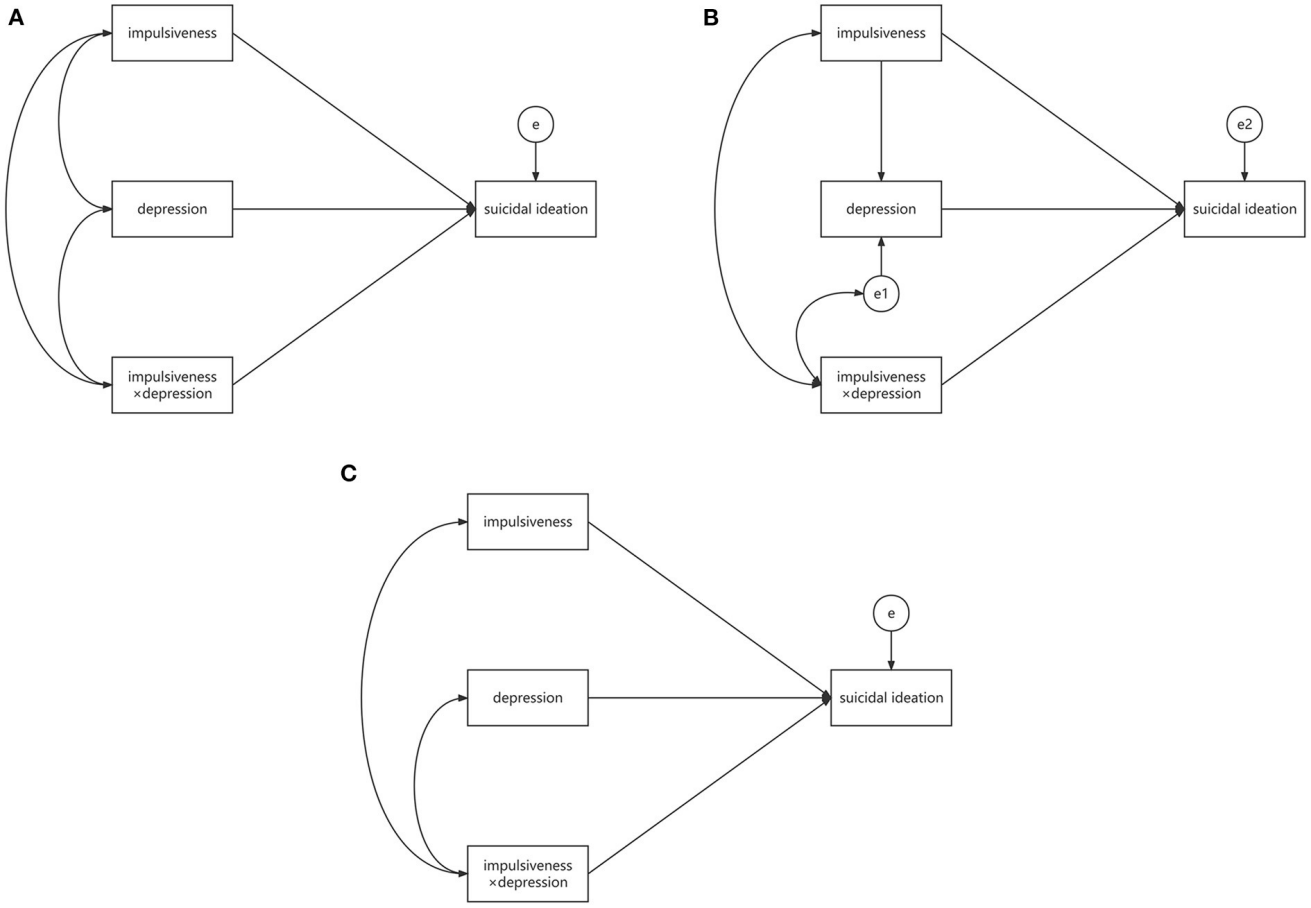
Statistical diagrams of baseline model **(A)**, MODMEDPM **(B)**, and SMODPM **(C)**. MODMEDPM—moderated mediation path model; SMODPM—simple moderation path model; “impulsiveness × depression”—the interaction term; “e”, “e1” and “e2”—the residual variances of dependent variables.

Firstly, we constructed a baseline model, which was a saturated model with all variables (impulsiveness, depression, interaction term of impulsiveness and depression, and suicidal ideation) linked by paths (statistical diagram shown in Figure 2A). Secondly, we chose MODMEDPM, which was defined Model 74 (statistical diagram shown in Figure 2B) in PROCESS macro (40), as the target model. Model 1 (statistical diagram shown in Figure 2C) in PROCESS (40), which was SMODPM, was chosen as the alternative model.

The indexes of chi-square value, Akaike information criterion (AIC), Bayesian information criterion (BIC), comparative fit index (CFI), Tucker & Lewis index (TLI) and root mean square error of approximation (RMSEA) were used to compare the model fits. The level of significant tests for the paths was set to 0.05. The mediating effect was examined with the bootstrap method with a sampling size of 5,000. The scores of the three variables, including impulsiveness, depression, and suicidal ideation, were mean centered before modeling.

## Results

### Correlation matrix of impulsiveness, depression, and suicidal ideation

Mean centered scores were used when doing correlation analyses. Given that the scores of SIOSS did not fit a normal distribution (Kolmogorov-Smirnov *Z* = 2.770, *p* < 0.001), we performed correlation matrix with spearman coefficients. The results showed that all the three variables were correlated with each other significantly, as shown in Table 2. The correlation matrix showed large correlation coefficients between depression and suicidal ideation (*r* = 0.726, *p* < 0.001), as well as between impulsiveness and depression (*r* = 0.633, *p* < 0.001). From the perspective of psychometrics, there are several items as observed indicators of the three variables presenting similar meanings, which may result in strong correlations of depression with impulsiveness and suicidal ideation. Considering the possible serious common method biases, we conducted a test and got the first common factor's explained variation as 24.31% [50%, as an empirical value in previous studies (41), was recommended the upper cut-off value of serious common method biases]. It meant that the common method biases in this study was tolerable. Therefore, the study results could illustrate the real relationships among variables.

**Table 2:** Correlation matrix of the scores of BIS, SDS and SIOSS.

		**BIS**	**SDS**	**SIOSS**
BIS	Spearman coefficient	1.000		
	*p*	N/A		
SDS	Spearman coefficient	0.633	1.000	
	*p*	<0.001	N/A	
SIOSS	Spearman coefficient	0.476	0.726	1.000
	*p*	<0.001	<0.001	N/A

BIS, Barratt impulsiveness scale; SDS, self-rating depression scale; SIOSS, self-rating idea of suicide scale.

### Model optimization

Although suicidal ideation scores did not fit normal distribution, its coefficients of skewness (=1.027) and kurtosis (=0.943) were lower than 2 and 7, respectively. According to Finney and DiStefano's estimating strategy for model parameters (42), maximum likelihood (ML) estimation could still be used as a robust method. In addition, the bootstrap method might help solve the non-normal distribution problem.

**Figure 3:**
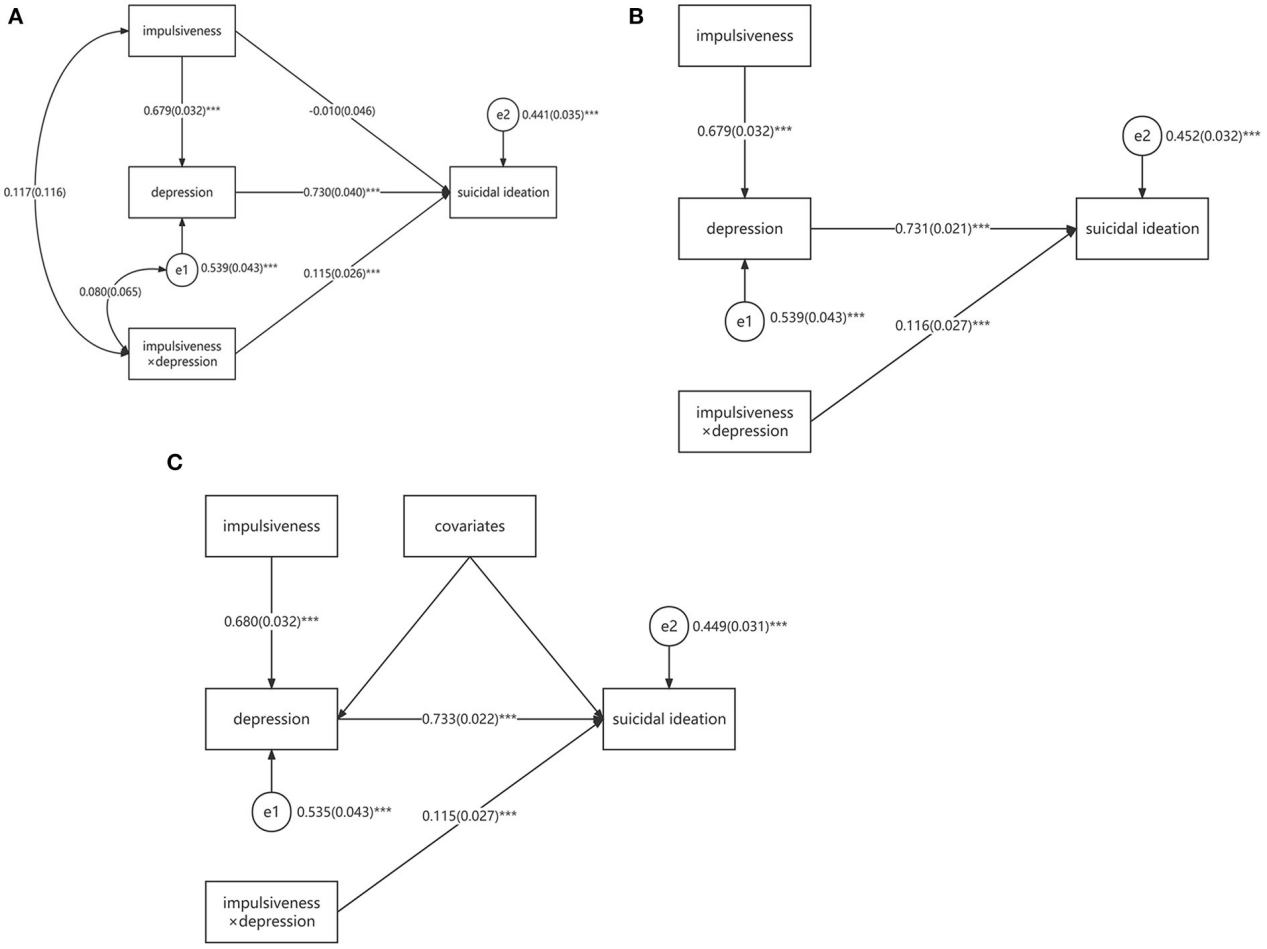
Statistical diagrams of saturated MODMEDPM [**(A)**, Model 2], relatively acceptable MODMEDPM [**(B)**, Model 3], and final MODMEDPM [**(C)**, Model 4]. ***p < 0.001; MODMEDPM—moderated mediation path model; “impulsiveness × depression”—the interaction term; “e,” “e1,” and “e2”—the residual variances of dependent variables; Covariates included gender, grade, residence, only child, family structure and age. The path coefficients were standardized.

The baseline model (Model 0) fit indexes showed that it was a saturated model with chi-square value and degrees of freedom equal to zero. Because moderation model hypothesized that the independent and moderating variable were not correlated with each other, we deleted the linkage between depression and impulsiveness and got the SMODPM (Model 1). However, the fit indexes of SMODPM were not good, as shown in Table 3. Then, we examined the target model MODMEDPM (Model 2). Model 2 was an algebraically equivalence model of Model 0 but was theoretically different. In Model 2, the path between impulsiveness and depression was unidirectional, while in Model 0, the path was bidirectional. Obviously, only Model 2 represented the mediation hypothesis. However, the fit indexes of Model 2 were the same with Model 0, which referred to a saturated model (see Table 3). The diagram and path coefficients were displayed in Figure 3. To optimize the model fit, we deleted the non-significant paths, namely, bidirectional path between impulsiveness × depression and impulsiveness (β = 0.117, SE = 0.116, *p* = 0.312), bidirectional path between impulsiveness × depression and e1 (β = 0.080, SE = 0.065, *p* = 0.219), and unidirectional path from impulsiveness to suicidal ideation (β = −0.010, SE = 0.046, *p* = 0.829). The Resulted Model 3 fitted well and could be accepted (indexes shown in Table 3). Finally, we included the demographic factors as covariates to control the confounding effects and resulted in Model 4, which fit better than Model 3 (see Table 3). None of the covariates had significant effect on dependent variables.

**Table 3:** Fit indexes of the models.

**Model**	χ^2^**/df**, ***p***	**RMSEA, 90%CI**	**CFI/TLI**	**AIC/BIC**
Model 0	0.000/0, 0.000	0.000, (0.000, 0.000)	1.000/1.000	15,914.878/15,973.311
Model 1	297.092/1, 0.000	0.785, (0.712, 0.862)	0.240/-1.279	16,209.971/16,264.230
Model 2	0.000/0, 0.000	0.000, (0.000, 0.000)	1.000/1.000	15,914.878/15,973.311
Model 3	9.842/3, 0.020	0.069, (0.024, 0.119)	0.990/0.983	15,918.720/15,964.632
Model 4	22.024/15, 0.107	0.031, (0.000, 0.057)	0.990/0.988	15,877.217/15,973.118

As recommended by Wen and Marsh (43), when sample size was between 250 and 500, the significant level for chi-square test was set to be 0.0005. The p value larger than 0.0005 indicated a good fit. Additionally, the recommended good fit standards of RMSEA, CFI and TLI were <0.08, >0.90, and >0.90, respectively. AIC and BIC were used to compare models, and lower values indicated a better fit. “χ^2^”, chi-square; “df”, degree of freedom; RMSEA, root mean square error of approximation; 90%CI, 90% confidence interval; CFI, comparative fit index; TLI, Tucker & Lewis index; AIC, Akaike information criterion; BIC, Bayesian information criterion.

### Moderated mediating effect analyses

#### Total effect

In the model, there were two nested models. Depression and suicidal ideation were the dependent variables in the two nested models, respectively. Depression was predicted by impulsiveness (R^2^ = 0.465, SE = 0.043, *p* < 0.001). Suicidal ideation was directly predicted by depression and indirectly predicted by impulsiveness. Meanwhile, the indirect effect was moderated by impulsiveness (R^2^ = 0.551, SE = 0.031, *p* < 0.001). This meant that the model could account for 55.1% of the total variances of suicidal ideation. The model summary was listed in Supplementary Table S1.

#### Mediating effect

As shown in Supplementary Table S2, the direct effect of impulsiveness on suicidal ideation was non-significant. The effects of impulsiveness on depression and of depression on suicidal ideation were significant [β_ondepression =_0.680, SE=0.032, 95%CI = (0.609, 0.737), β_onsuicidalideation =_0.733 SE = 0.022, 95%CI = (0.688, 0.773)], and the indirect effect of impulsiveness on suicidal ideation was significant [β_indirect_ = 0.499, SE = 0.031, 95%CI = (0.436, 0.558)]. The indirect effect here could be explained as mediating effect because all the significant coefficients were positive.

#### Moderating effect

**Figure 4:**
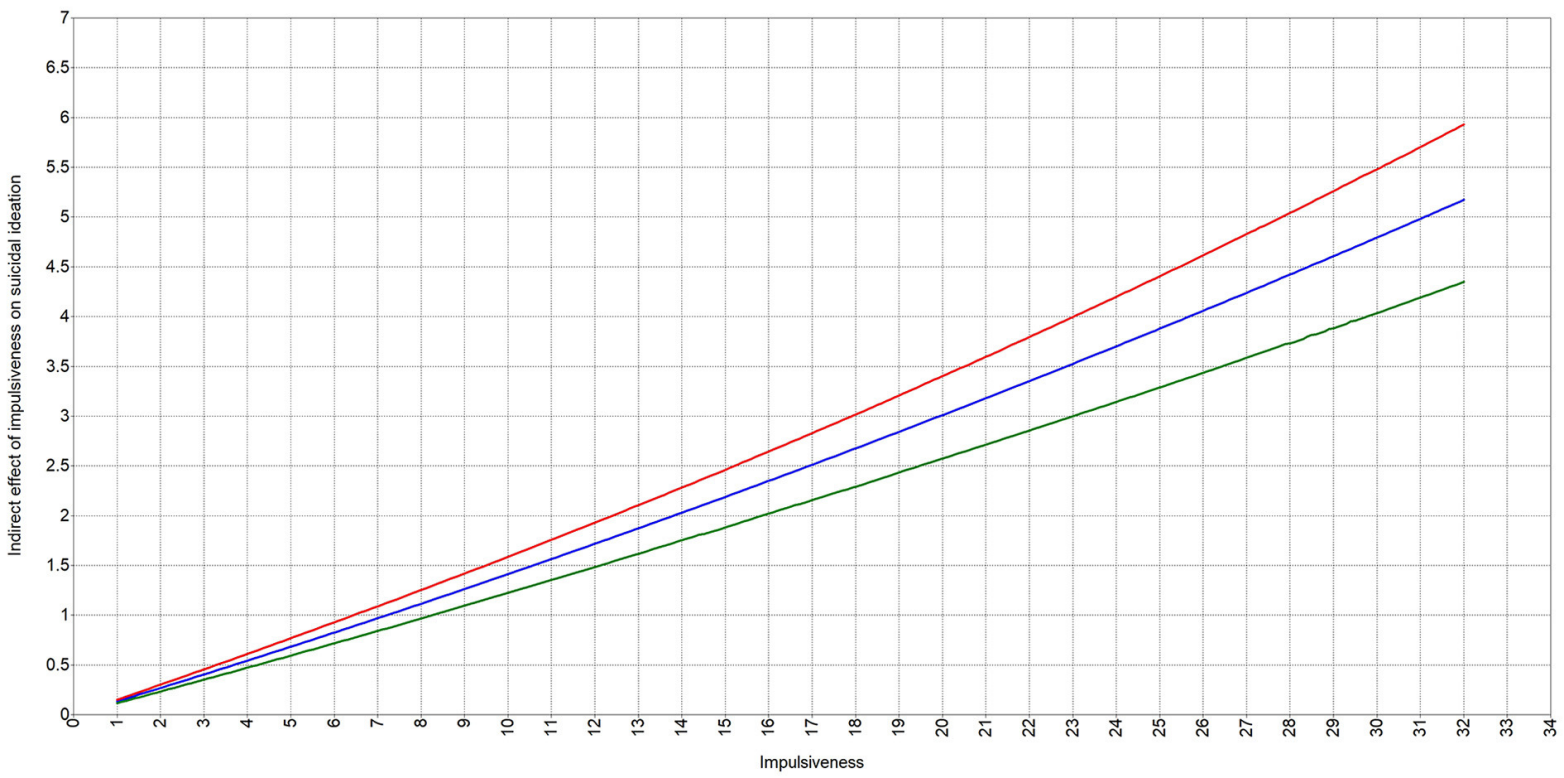
Simple slope diagram. Red line—the indirect effect curve under condition of high impulsiveness; Blue line—the indirect effect curve under condition of medium impulsiveness; Green line—the indirect effect curve under condition of low impulsiveness.

We regarded the coefficient of path from the interaction term to suicidal ideation as the effect parameter of moderation [β_moderation =_0.115, SE = 0.027, 95%CI = (0.059, 0.167)]. However, this coefficient really represented the moderating effect on the path from depression to suicidal ideation. To test the moderating effect of impulsiveness on the indirect effect from impulsiveness to suicidal ideation, the conditional indirect effect analysis (simple slope analysis) was conducted. Firstly, impulsiveness was divided into three conditions: low impulsiveness (mean-1SD), medium impulsiveness (mean), and high impulsiveness (mean + 1SD). Secondly, we calculated the simple slopes, which represented the moderated mediating effects, under the three conditions, respectively. Thirdly, we compared the three slopes to define whether they were statistically different. As these were multiple comparisons, the significant level was set to be 0.05/3 = 0.017. The results showed that the slope under the condition of high impulsiveness was higher than that under the condition of medium and low impulsiveness (ΔSlope_High−Low_ = 0.028, SE = 0.008, *p* < 0.001; ΔSlope_High−Medium_ = 0.014, SE = 0.004, *p* < 0.001), and the slope under the condition of medium impulsiveness was higher than that under the condition of low impulsiveness (ΔSlope_Medium−Low_ = 0.014, SE = 0.004, *p* < 0.001, see Supplementary Table S3). The simple slope diagram was shown in Figure 4. It indicated that impulsiveness positively moderated the indirect effect of impulsiveness on suicidal ideation. In other words, the mediating effect of depression between impulsiveness and suicidal ideation was reinforced by impulsiveness.

## Discussion

In the present study, we found that the moderated mediation path model could finely describe the complex relationships among impulsiveness, depression, and suicidal ideation. Impulsiveness could indirectly lead to suicidal ideation through depression and enhance the indirect effect. These findings confirmed the hypothesis. Additionally, we found that impulsiveness did not affect suicidal ideation directly nor independently, and the presence of depression was necessary as both mediating and interacting variable.

Although impulsiveness was correlated with suicidal ideation in correlation matrix, the direct path from impulsiveness to suicidal ideation was not significant in the model. In a word, it can be explained as the complete mediating effect. This indicates that impulsiveness probably predicts depression and depression sequentially predicts suicidal ideation. It partly illustrates the mechanism of impulsiveness' effect on suicidal ideation. Impulsiveness in motivational-volitional model is not among the risk factors for suicidal ideation (5). This theoretical hypothesis was verified in model-based studies in which depression was included (13, 14), which might be due to the strong mediating effect. Therefore, conclusion can be drawn that impulsiveness is an indirect risk factor for suicidal ideation. We had noticed that the coefficient of the direct path was negative. In some studies (44–46), this kind of indirect effect is named suppressing effect. Nevertheless, it is based on the condition that the coefficient is significant. In the present study, the coefficient of direct effect from impulsiveness to suicidal ideation was far away from significance (there was 82.9% of probability for us to accept the null hypothesis). We tend to regard it as random error rather than a real effect.

The mediating effect indicates that depression is an important bridge between impulsiveness and suicidal ideation. On one hand, depression is a vital cause for suicidal ideation, which has been proved in previous studies, but with different interpretations [e.g., hopelessness (47), psychological pain (48), impaired executive function (49), and lack of pleasure (50, 51)]. On the other hand, depression is proved related to impulsiveness (20, 52, 53). In the present study, impulsiveness was scaled as a trait, thus logically pre-existed compared to depression. There was also a study confirming that depression was an outcome of impulsiveness, in which longitudinal designed observation was applied (54). Therefore, impulsiveness predicts depression. The bridge role of depression does not agree with the clinical diathesis-stress model (9), in which depression interacts with impulsiveness. It may not be reasonable to regard depression as stress itself, but a consequence when stress occurred, as is hypothesized in Beck's cognitive model (4).

Simultaneously, impulsiveness is the moderator for the mediating effect of impulsiveness on suicidal ideation through depression. Previous studies (55, 56) has discussed the moderating effect of impulsiveness between depression and suicidal ideation, finding that high level of impulsiveness can increase the depression's effect on suicidal ideation, which agrees with the present study. However, they do not consider the correlation between impulsiveness and depression, which exists and can lead to biases in the moderating effect analyses. Combining these two kinds of effects, we construct the well-fitted moderated mediating model, and illustrate the importance of both impulsiveness and depression in the generating of suicidal ideation. Depression is the necessary mediator between impulsiveness and suicidal ideation. Without depression, impulsiveness may not generate suicidal ideation directly. The precondition of impulsiveness leading to depression may be the existence of stress, in accordance with Beck's cognitive model of suicide (4). Impulsiveness is the reinforcer of its indirect effect on suicidal ideation. Therefore, impulsiveness is vital in generating of suicidal ideation, although it does not contribute to this process independently nor directly.

Some previous studies have proved that age is an important factor of impulsiveness (57, 58). Compared to the adults, adolescents are more impulsive (57, 58). The underlie mechanism may be the “ongoing maturation of parietal brain areas in adolescents” (58). Therefore, age's influence on impulsiveness may be the result of brain development. In the present study, to control the possible confounding effects of age, we only recruited participants who are adolescents and young adults aged from 16 to 21. According to the previous studies (57, 58), they might be more susceptible to impulsiveness than older adults. It is not clear whether the present results can be generalized to individuals of other age groups, which needs more considerations in future studies.

There are still some limitations of this study. First, we do not consider stress in this model, which is probably the moderator of impulsiveness' effect on depression and suicidal ideation. Second, the cross-sectional design cannot interpret the real causal relationships among impulsiveness, depression, and suicidal ideation. We only construct the unidirectional path model based on theoretical analyses. In fact, there are studies (59, 60) show that depression as a trait (e.g., the depressive component of affective temperament) can also predict suicide behaviors. This indicates that depression is not always the outcome of impulsiveness but may overlap with it on the trait level. What roles impulsiveness and depression play in generating suicidal thoughts and behaviors needs more evidence from longitudinal studies. Third, the sample comes from college students. It is not clear whether the results could be generalized to a more widely range of populations. Future studies would include both stress and depressive trait as the predictors and conduct longitudinal design to optimize the model. Meanwhile, more participants of different age, social status, occupations, and ethnics can be included for unbiased results.

## Conclusions

The present study finds that impulsiveness indirectly affects suicidal ideation through the mediator depression, and simultaneously moderates the mediating effect. This implies first that people who have impulsive traits are riskier to generate suicidal thoughts because they are more likely to suffer from depression. Second, people who are depressive have even higher risk to develop suicidal thoughts when they simultaneously have impulsive traits. Therefore, in clinical and health care work, when considering depression to prevent suicidal ideation, impulsiveness needs to be monitored throughout the process of premorbid and onset stages of depression.

## Data availability statement

The raw data supporting the conclusions of this article will be made available by the authors, without undue reservation.

## Ethics statement

The studies involving human participants were reviewed and approved by the Medical Ethics Committee of Army Medical University. Written informed consent to participate in this study was provided by the participants' legal guardian/next of kin.

## Author contributions

JZ contributed to the design of the research, conducting statistical analyses, and drafting the manuscript. XZ contributed to the arranging of materials and data, conducting statistical analyses, and editing manuscript. GY contributed to the data processing and manuscript revising. ZF contributed to the design of the whole study and critical revising of the manuscript. All authors have read and revised the final manuscript. All authors contributed to the article and approved the submitted version.

## Funding

This research is supported by the National Natural Science Foundation of China (81971278).

## Conflict of interest

The authors declare that the research was conducted in the absence of any commercial or financial relationships that could be construed as a potential conflict of interest.

## Publisher's note

All claims expressed in this article are solely those of the authors and do not necessarily represent those of their affiliated organizations, or those of the publisher, the editors and the reviewers. Any product that may be evaluated in this article, or claim that may be made by its manufacturer, is not guaranteed or endorsed by the publisher.

## Abbreviations

UPPS: urgency-premeditation-perseverance-sensation-seeking
MODMEDPM: moderated mediation path model
SMODPM: simple moderation path model
SDS: self-rating depression scale
BIS: Barratt impulsiveness scale
SIOSS: self-rating idea of suicide scale
RMSEA: root mean square error of approximation
CFI: comparative fit index
TLI: Tucker & Lewis index
AIC: Akaike information criterion
BIC: Bayesian information criterion
SD: standard deviation
ML: maximum likelihood
SE: standard error
CI: confidence interval.
