# Supplementary material for: Impulsiveness indirectly affects suicidal ideation through depression and simultaneously moderates the indirect effect: A moderated mediation path model
Source: Front Psychiatry. 2022 Jul 27;13:913680. doi: 10.3389/fpsyt.2022.913680 (PMC9363579; doi:10.3389/fpsyt.2022.913680)
Supplement: Supplementary file 1 [file Table_1.doc]

Table 1S Model summary information of final MODMEDPM

|  | **Consequent** | | | | | | |
| --- | --- | --- | --- | --- | --- | --- | --- |
|  | **Depression (n=480)** | | | | **Suicidal ideation (n=480)** | | |
| **Statistics** | **Estimate** | **SE** | ***p*** | **Estimate** | | **SE** | ***p*** |
| R^2^ | 0.465 | 0.043 | <0.001 | 0.551 | | 0.031 | <0.001 |
| RESID | 0.535 | 0.043 | <0.001 | 0.449 | | 0.031 | <0.001 |

Note: MODMEDPM – moderated mediation path model; R^2^ – variances that could be explained by the model; RESID – residual variances (standardized); SE – standard error.

Table 2S Effect coefficients of final MODMEDPM

|  | **Consequent** | | | | | | | | |
| --- | --- | --- | --- | --- | --- | --- | --- | --- | --- |
|  | **Depression (n=480)** | | | | **Suicidal ideation (n=480)** | | | | |
| **Variables** | **Estimate** | **SE** | ***p*** | **95%CI** |  | **Estimate** | **SE** | ***p*** | **95%CI** |
| Depression |  |  |  |  |  | 0.733 | 0.022 | <0.001 | [0.688, 0.773] |
| Impulsiveness | 0.680 | 0.032 | <0.001 | [0.609, 0.737] | (Indirect effect) | 0.499 | 0.031 | <0.001 | [0.436, 0.558] |
| Depression×Impulsiveness |  |  |  |  |  | 0.115 | 0.027 | <0.001 | [0.059, 0.167] |
| Gender | 0.068 | 0.070 | 0.325 | [-0.071, 0.203] |  | -0.086 | 0.065 | 0.184 | [-0.213, 0.042] |
| Grade | 0.004 | 0.086 | 0.965 | [-0.168, 0.170] |  | 0.044 | 0.077 | 0.567 | [-0.114, 0.190] |
| Residence | 0.070 | 0.088 | 0.425 | [-0.100, 0.243] |  | -0.026 | 0.082 | 0.750 | [-0.190, 0.128] |
| Only-child | -0.028 | 0.076 | 0.712 | [-0.173, 0.119] |  | 0.049 | 0.070 | 0.485 | [-0.084, 0.189] |
| Family structure | -0.005 | 0.069 | 0.938 | [-0.137, 0.132] |  | 0.009 | 0.053 | 0.868 | [-0.093, 0.117] |
| Age | -0.001 | 0.052 | 0.980 | [-0.106, 0.098] |  | -0.004 | 0.044 | 0.924 | [-0.097, 0.081] |

Note: MODMEDPM – moderated mediation path model; SE – standard error; 95%CI – 95% confidence interval; The path coefficients were standardized.

Table 3S Simple slope statistics (non-standardized) and significance tests

| **Condition** | **Slope/ΔSlope** | **SE** | ***p*** | **95%CI** |
| --- | --- | --- | --- | --- |
| Low impulsiveness | 0.118 | 0.009 | <0.001 | [0.101, 0.136] |
| Medium impulsiveness | 0.132 | 0.009 | <0.001 | [0.115, 0.149] |
| High impulsiveness | 0.146 | 0.010 | <0.001 | [0.125, 0.164] |
| High-Low | 0.028 | 0.008 | <0.001 | [0.014, 0.044] |
| High-Medium | 0.014 | 0.004 | <0.001 | [0.007, 0.022] |
| Medium-Low | 0.014 | 0.004 | <0.001 | [0.007, 0.022] |

Note: ΔSlope – the difference of slope between two conditions; SE – standard error; 95%CI – 95% confidence interval; In these multiple comparisons, the p value lower than 0.05/3=0.017 represented statistically significant.
